# Inhibitory effect of sodium houttuyfonate on synovial proliferation *in vitro* in cells from a patient with rheumatoid arthritis

**DOI:** 10.3892/etm.2014.1636

**Published:** 2014-03-27

**Authors:** JUN LI, TING ZHOU, FUTAO ZHAO

**Affiliations:** 1 Department of Rheumatology and Immunology, Shanghai Third People’s Hospital, School of Medicine, Shanghai Jiao Tong University, Shanghai 201999, P.R. China; 2 Department of Rheumatology and Immunology, Huainan First People’s Hospital, Huainan, Anhui 232000, P.R. China

**Keywords:** rheumatoid arthritis, sodium houttuyfonate, synovial proliferation, MTT assay

## Abstract

The aim of the present study was to investigate the inhibitory effect of sodium houttuyfonate (SH) on synovial cell proliferation *in vitro*. Primary cells were obtained from the synovial tissue of a patient with rheumatoid arthritis (RA). The cells were divided into five treatment groups as follows: the control group (group 1), 25 μg/ml SH-treated group (group 2), 50 μg/ml SH-treated group (group 3), 100 μg/ml SH-treated group (group 4) and the 200 μg/ml SH-treated group (group 5). Following seven days of treatment, the proliferation rate of the synovial cells was then detected using an MTT assay. The expression level of proliferative synovial cells markedly decreased in the SH-treated groups in a dose-dependent manner compared with the control group. In conclusion, the present study demonstrated that SH was able to inhibit the proliferation of synovial cells obtained from a patient with RA. These results provide a potential theoretical basis for the development of a safe and effective treatment against RA in the future.

## Introduction

Rheumatoid arthritis (RA) is the most common type of inflammatory arthritis, affecting between 0.5 and 1% of the population worldwide, regardless of geographical location and ethnicity (1,2). Although the etiology of RA remains to be fully elucidated, numerous studies have suggested that a combination of environmental and genetic factors are responsible. However, although environmental and genetic factors have been demonstrated to be important, they are insufficient for full expression of the disease. The primary inflammatory site in RA is the synovium. The proliferation of synovial fibroblasts is one of the main characteristics of RA and is considered to be necessary for the initiation and long-term progression of joint destruction in RA (3–6). In the long-term, joint destruction may lead to limited functions, decreased work ability and, more importantly, a decrease in the quality of life for patients with RA. In addition, RA is associated with an increased risk of cardiovascular disease and, thus, the life expectancy of patients with RA may be reduced by 3–18 years (7). Therefore, the inhibition of synovial hyperplasia during the early stages of disease progression may provide a potential therapeutic approach for the treatment of RA.

*Houttuynia cordata* Thunb (HCT) is a perennial herbaceous plant that grows in the wild in moist, shady areas in numerous Asian countries, including India. HCT has been widely used in China, Japan and other Asian countries as a medicine due to its anti-inflammatory properties (8). Previous studies have demonstrated that HCT has anti-inflammatory effects in a wide range of diseases (8–11). During the outbreak of hand, foot and mouth disease (HFMD) in 2008 in China, HCT was used as a therapeutic drug (12). Several studies demonstrated that HCT inhibited enterovirus 71 and coxsackievirus A16, which are the two main causative agents of HFMD (13,14). It has also been demonstrated that HCT water extract was able to treat severe acute respiratory syndrome (8,15,16) and herpes simplex virus infection (17). Sodium houttuyfonate (SH), an addition compound of sodium bisulfite and houttuynia, is the stable form of houttuynia and exhibits the same effect as HCT. Certain studies have demonstrated that SH has an antibacterial effect against 21 strains of *Staphylococcus aureus* (18) and it has previously been used for the treatment of cationic bovine serum albumin-induced membranous glomerulonephritis in BALB/c mice (19). Previous studies have also demonstrated that SH exerts an anti-inflammatory effect by inhibiting the tumor necrosis factor-α (TNF-α) pathway (20), which led to the hypothesis that SH may also be effective for the treatment of RA.

## Materials and methods

### Materials

Synovial tissue was obtained from a patient at the Department of Orthopedics and Pathology, Shanghai Third People’s Hospital (Shanghai, China). SH was purchased from Shanghai Qingping Pharmaceutical Co., Ltd. (Shanghai, China; batch number 0701-3). D-Hank’s solution and RPMI-1640 nutrient medium were provided by the laboratory of the Shanghai Third People’s Hospital, School of Medicine, Shanghai Jiao Tong University (Shanghai, China). Fetal bovine serum (FBS) was purchased from Beijing Ruizekang Biotech Co., Ltd. (Beijing, China). Type II collagenase and trypsin were obtained from Shanghai Qifa Experimental Reagent Co., Ltd. (Shanghai, China). The MTT kit was purchased from Sigma (St. Louis, MO, USA). Informed consent was obtained from the patient.

### Cultivation of primary cells from a patient with RA

**Figure 1:**
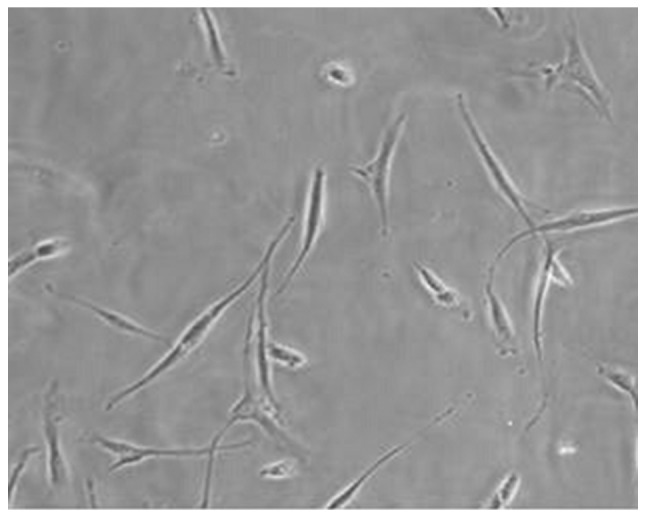
Primary synovial cells obtained from a patient with rheumatoid arthritis (magnification, ×100).

The fat and fibrous tissue was removed from the synovial tissue. The tissue was then washed three times with D-Hank’s solution and cut into two sections (1–2 mm in size). The tissue was placed in 25 cm^2^ culture bottles containing 2 ml RPMI-1640 nutrient solution in 10% FBS and 2 ml 0.4% type II collagenase. The culture bottles were incubated at 37°C and 5% CO_2_ for 2 h. Unattached cells were then transferred into centrifuge tubes and centrifuged for 10 min. A total of 4 ml 0.25% trypsin was added and the cells were incubated for 30 min. The solution was then filtered using a 200-mesh nylon net. Following centrifugation for 10 min, the cells were incubated as aforementioned for 24 h. The unattached cells were discarded, leaving primary cells from a patient with RA (Fig. 1).

### Experimental groups and administration

The primary cells were equally divided into five groups as follows: the control group (group 1), cells treated with 25 μg/ml SH (group 2), 50 μg/ml SH (group 3), 100 μg/ml SH (group 4) and 200 μg/ml SH (group 5). Group 1 was administered an equivalent amount of normal saline (NS), whilst groups 2 to 5 were treated with corresponding amounts of SH. NS and SH were administered daily for 7 days by transfer pipette. Following the final administration, the five groups of synovial cells were measured using an MTT assay for analysis of the growth inhibitory rate of SH on synovial proliferation.

### MTT assay

Sequential dilutions of cells in the culture medium between 10^6^ and 10^3^ cells/ml were prepared. A total of 100 μl each dilution was analyzed in triplicate, using a microplate reader (Bio-Rad, Hercules, CA, USA) and three control wells containing medium only were used as an absorbance reference. The cells were then incubated for 24 h. A total of 10 μl MTT reagent (0.25% MTT) was added to each well and the cells were further incubated for 4 h until a purple precipitate was observed. A total of 100 μl detergent reagent was added to each well and swirled gently, and the plate was then incubated in the dark overnight at room temperature. The absorbance in each well at 570 nm was measured using a microplate reader. Finally, the data were recorded and the results were analyzed.

### Data interpretation and statistical analysis

If the absorbance rate was lower than the control, this was considered to indicate a reduction in cell proliferation. By contrast, if the absorbance rate was higher, this indicated an increase in cell proliferation. The difference in the inhibition rate between the groups was analyzed using one-way analysis of covariance. P<0.05 was considered to indicate a statistically significant difference.

## Results

### Inhibition rate of SH on synovial proliferation in cells from a patient with RA

**Figure 2:**
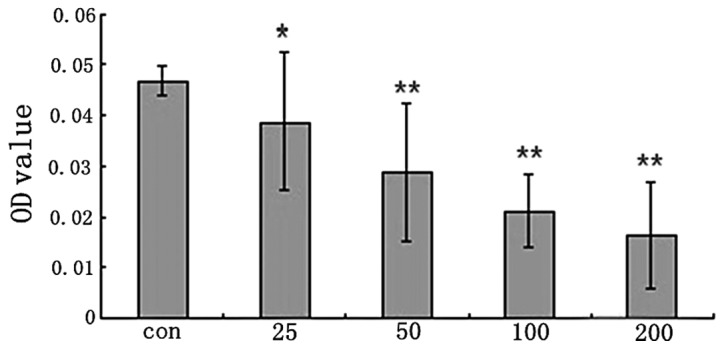
Inhibitory effect of SH on the proliferation of synovial cells from a patient with rheumatoid arthritis. The cells were treated with 25, 50, 100 and 200 μg/ml SH. ^*^P<0.05 and ^**^P<0.01 versus con. OD, optical density; con, control; SH, sodium houttuyfonate.

**Figure 3:**
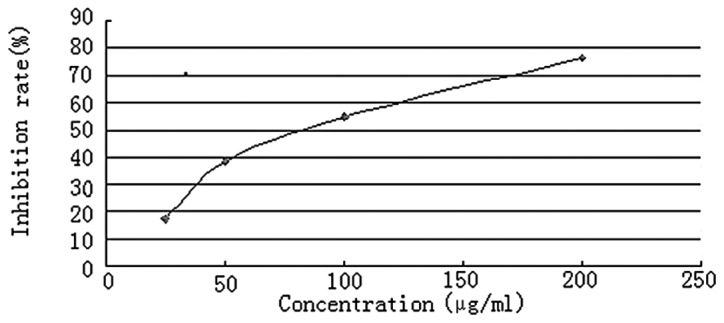
Inhibition rate of different concentrations of sodium houttuyfonate.

As shown in Fig. 2, the proliferation rate of synovial cells was markedly higher in the control group compared with the other groups (P<0.05). In the SH-treated groups, the proliferation rates of synovial cells were significantly decreased compared with those in group 1 (P<0.05 for group 2; P<0.01 for groups 3–5). These results indicated that SH decreased the level of synovial proliferation in cells from a patient with RA. Fig. 3 shows the inhibition rate of different concentrations of SH on synovial proliferation. The inhibition rates of different concentrations of SH are shown numerically in Table I and the correlation coefficient was found to be 0.961718.

## Discussion

The proliferation of synovial fibroblasts leads to the development of RA and initiates joint destruction in the long term (3–5). Previous studies have demonstrated that TNF-α, interleukin (IL)-6 and IL-8 are important proinflammatory cytokines in the pathogenesis of RA (21–24), and that the inhibition of TNF-α and IL-6 is effective in the treatment of patients with RA (25–27). Notably, several studies have indicated that HCT was able to efficiently inhibit IL-6, IL-8 and TNF-α (24,28). In addition, nonsteroidal anti-inflammatory drugs (NSAIDs) have been demonstrated to be efficacious in the treatment of RA by binding to cyclooxygenase (COX) enzymes and therefore inhibiting the production of prostaglandins. Previous studies also demonstrated that HCT supercritical extract exerted an anti-inflammatory effect by inhibiting the COX-2/prostaglandin E2 pathway. Owing to the gastrointestinal side-effects of NSAIDs, HCT may be a better drug candidate for the alleviation of symptoms caused by RA (20). The present study demonstrated that the synovial proliferation rate significantly decreased following treatment with SH. Furthermore, the inhibition rate of SH was found to be dose dependent. Therefore, these results suggest that SH is able to inhibit the proliferation of synovial cells from a patient with RA.

The results from the present study provide a potential theoretical basis for the treatment of RA. Furthermore, due to the dose-dependent reaction of SH observed in the present study, a suitable dose of SH may almost completely inhibit synovial proliferation and therefore be highly effective in the clinical treatment of patients with RA.

## Figures and Tables

**Table I:** Association between the inhibition rate of sodium houttuyfonate and its concentration. The correlative coefficient is 0.961718.

Inhibition rate (%)	Concentration (μg/ml)
17.02	25
38.30	50
54.79	100
76.60	200
